# Total wrist arthroplasty efficacy and safety: a systematic review

**DOI:** 10.1016/j.jor.2026.04.001

**Published:** 2026-04-10

**Authors:** Gaetano Pappalardo, Luise Schäfer, Domenico Falco, Nicola Tammaro, Jörg Eschweiler, Filippo Migliorini

**Affiliations:** a Department for Orthopaedic and Trauma Surgery, Pineta Grande Hospital, Castel Volturno, Italy; b Department of Orthopaedic and Trauma Surgery, Eifelklinik St.Brigida, Kammerbruschstr. 8, 52152, Simmerath, Germany; c Department of Trauma and Reconstructive Surgery, University Hospital of Halle, Martin-Luther University Halle-Wittenberg, 06097, Halle (Saale), Germany; d Department of Orthopaedic and Trauma Surgery, Academic Hospital of Bolzano (SABES-ASDAA), via Lorenz Böhler 5, 39100, Bolzano, Italy; e Department of Life Sciences, Health, and Health Professions, Link Campus University, Via del Casale di San Pio V, 00165, Rome, Italy; f Department of Trauma and Reconstructive Surgery, BG Klinikum Bergmannstrost Halle GmbH, Halle (Saale), Germany

**Keywords:** Wrist arthrosis, Total wrist arthroplasty, TWA

## Abstract

**Introduction:**

Wrist arthrosis is a clinically relevant condition with considerable impact on patients’ daily lives. While total wrist arthroplasty (TWA) has emerged as a motion-preserving alternative to arthrodesis, evidence regarding its long-term effectiveness and safety remains heterogeneous. Therefore, this study systematically reviews clinical outcomes of TWA, including patient-reported outcome measures (PROMs), functional performance, and surgical safety endpoints such as complications, revisions, and reoperations.

**Methods:**

The present systematic review followed the guidelines defined in the 2020 Preferred Reporting Items for Systematic Reviews and Meta-Analyses (PRISMA) statement. PubMed, Web of Science, and Embase were accessed in January 2025 without additional filters or temporal constraints. All clinical studies investigating the clinical outcomes of TWA were included. Data concerning the visual analogue scale (VAS), disability of arm, shoulder, and hand (DASH), patient-reported wrist evaluation (PRWE) score, grip strength and ROM were collected at baseline and at last follow-up. Data on complications, reoperation and revision rates were collected postoperatively. Only studies with a minimum of one year of follow-up were included. The methodological quality of the included studies was assessed using the ROBINS-I tool for non-randomised studies.

**Results:**

Data on 2503 wrists from 2362 patients across 62 studies were retrieved. All PROMs and functional tests showed a statistically significant improvement from baseline to the last follow-up (p < 0.001 for each parameter). A total of 924 postoperative complications were reported over 2503 operated wrists (36.9%). 16.3% (408 of 2503) wrists underwent revision, and 18.4% (461 of 2503) needed further surgical interventions because of complications.

**Conclusion:**

TWA appears to be effective in improving pain and disability scores for patients affected by wrist arthrosis. However, some uncertainty persists regarding the safety profile of the surgical procedure. Future studies should focus on reducing postoperative complications.

**Level of evidence:**

Level II

## 1 Introduction

Wrist arthrosis represents a significant source of pain and functional disability, which commonly affects patients with degenerative osteoarthritis, post-traumatic, inflammatory, and metabolic conditions 1, 2, 3. The prevalence of wrist osteoarthritis has been increasing, given the ageing population and improved long-term survival of people with inflammatory arthropathies, such as rheumatoid arthritis 4, 5, 6. Frequently, patients complain of limitations in activities of daily living, such as personal hygiene, feeding, work-related activities and private entertainment, marking a reduced quality of life. ^3^^,^^7^^,^^8^ Historically, total wrist fusion (TWF) was initially considered the gold standard in the surgical management of severe symptomatic wrist pathology thanks to its pain relief and mechanical stability. ^2^^,^^9^^,^^10^ However, TWF sacrifices wrist mobility, significantly impacting daily activities, especially in young and active patients 11, 12, 13. Total wrist arthroplasty (TWA) represents a motion-preserving alternative, with the aim to alleviate pain and simultaneously maintain the functional ROM (range of motion) and kinematics. ^3^^,^^9^ Over the last decades, several wrist prosthesis designs have been developed, with the primary goal of enhancing functional wrist recovery, improving implant longevity, and reducing implant-associated complications. ^3^^,^^14^ Early models, such as the BIAX prosthesis, were associated with high rates of implant loosening, mechanical failure, and revision surgery, which limited their use and progressive adoption 15, 16, 17. Subsequently, cemented and uncemented designs were developed to optimise stability and promote osseointegration. Initially, cemented implants afforded a better postoperative stability but did not prevent long-term loosening. ^18^^,^^19^ On the other hand, uncemented designs often faced challenges related to insufficient bone ingrowth and stress shielding 20, 21, 22. Innovation in implant biomechanics and geometry, fixation techniques, and material optimisation has led to the modern fourth generation of implants, such as the Universal 2, ReMotion, and Maestro systems. ^2^^,^^23^ Those implants optimise load distribution, maintain joint kinematics, and reduce the likelihood of revision, although their safety and long-term efficacy remain under investigation. ^2^^,^^9^^,^^24^ While single-centre studies and multicenter cohorts report improvements in pain, disability and functional scores with modern wrist prosthesis, complication rates such as implant loosening, periprosthetic fracture, and infection remain substantial and up to 18% requiring revision or additional surgery. ^9^^,^^23^^,^^25^^,^^26^ The performance and clinical use of different prosthetic designs, and their influence on long-term outcomes, remain unclear. For surgical planning and patient counselling, it is essential to understand these differences among implants, particularly with respect to the nuances between functional preservation and procedural risks ^16^^,^^27^, ^28^, ^29^, ^30^, ^31^. Patient-reported outcome measures (PROMs) and functional assessments have gained importance in evaluating TWA success and indications, offering insights into how the surgical procedure improves quality of life, grip strength, ROM, and daily function. ^3^^,^^8^^,^^32^^,^^33^ The literature on postoperative outcomes remains heterogeneous and unclear, whereas prosthetic wrist technology has improved in quality. An evaluation of efficacy and safety is essential for guiding evidence-based clinical decision-making and defining gaps that may influence future research priorities and directions ^1^^,^^34^, ^35^, ^36^, ^37^. By analysing data from studies that include diverse patient populations and implant designs, the present study aims to investigate the effectiveness and safety profile of TWA by evaluating PROMs, functional tests, and complication, revision, and reoperation rates.

## 2 Methods

### 2.1 Eligibility criteria

All clinical studies that investigated the clinical outcomes of TWA were considered. According to the authors’ capabilities, only articles in the following languages were considered: English, German, French, Spanish, and Italian. According to the Oxford Centre for Evidence-Based Medicine, ^29^ studies with levels of evidence I to III were eligible. Eligible studies were required to be published in peer-reviewed journals. Exclusion criteria comprised reviews, editorials, letters, opinions, studies involving in vitro experiments, biomechanical assessments, computational analyses, animals, or cadaveric research. Only clinical trials reporting on the outcomes of primary TWA were included. Studies on revision-TWA were disregarded. Only studies with a minimum of one year of follow-up were included.

### 2.2 Search strategy

The present systematic review followed the guidelines defined in the 2020 Preferred Reporting Items for Systematic Reviews and Meta-Analyses (PRISMA) statement. ^30^ The following framework was used for the search:

• P (Problem): wrist arthrosis;
• I (Intervention): TWA;
• O (Outcomes): PROMs, functionality tests, and complication, revision, and reoperation rates;
• T (Timing): minimum one year of follow-up;
• D (Design): clinical trials.

In January 2025, PubMed, Web of Science, and Embase were accessed without additional filters or temporal constraints. The Medical Subject Headings (MeSH) used in the database search are reported in Table 1.

**Table 1:** Strings used for the search in each database.

Database	Search Strategy (MeSH terms and keywords)
PubMed	(“Arthroplasty, Replacement, Wrist”[MeSH Terms] OR “total wrist arthroplasty” OR “total wrist replacement” OR TWA) AND (“efficacy” OR “safety” OR “complications” OR “revision” OR “reoperation” OR “outcome∗” OR “follow-up”)
Web of Science	TS=(“total wrist arthroplasty” OR “total wrist replacement” OR TWA) AND TS=(“efficacy” OR “safety” OR “complications” OR “revision” OR “reoperation” OR “outcome∗” OR “follow-up”)
Google Scholar	“total wrist arthroplasty” AND (“efficacy” OR “safety” OR “complications” OR “revision” OR “reoperation” OR “outcome∗” OR “follow-up”)
Embase	(‘total wrist arthroplasty’/exp OR ‘total wrist replacement’ OR TWA) AND (‘efficacy’ OR ‘safety’ OR ‘complications’ OR ‘revision’ OR ‘reoperation’ OR ‘outcome∗’ OR ‘follow-up’)

### 2.3 Data items

Data extraction was conducted by two authors (∗∗; ∗∗). The following generalities were collected for each study: author, year of publication, journal, prosthesis model and manufacturer, and length of follow-up. The following baseline data were retrieved: number of patients, number of women, number of wrists, and mean age. Data concerning the visual analogue scale (VAS), disability of arm, shoulder, and hand (DASH), patient-reported wrist evaluation (PRWE) score, grip strength and ROM were collected at baseline and at last follow-up. Data on complications, reoperation and revision rates were collected postoperatively. Extraction was performed using Microsoft Office Excel version 16.0 (Microsoft Corporation, Redmond, USA).

### 2.4 Methodology quality assessment

The methodological quality assessment was performed by two authors (∗∗; ∗∗). The Risk of Bias in Nonrandomised Studies of Interventions (ROBINS-I) tool ^38^ was employed to evaluate non-randomised controlled trials (non-RCTs). This tool assesses seven domains of potential bias in non-RCTs, including domains focusing on possible confounding factors and patient selection characteristics before the comparative intervention, bias in classification during the intervention, as well as methodological quality post-intervention comparison, which involves deviations from previously intended interventions, missing data, erroneous outcome measurement, and bias in reported outcome selection. The ROBINS-I chart was generated using the Robvis Software (Risk-of-bias VISualization, Riskofbias.info, Bristol, UK). ^39^

### 2.5 Synthesis methods

The statistical analyses were performed by the main author (∗∗) following the recommendations of the Cochrane Handbook for Systematic Reviews of Interventions. ^37^ For descriptive statistics, the IBM SPSS software version 25 (International Business Machines Corporation, Armonk, USA) was used. The arithmetic mean and standard deviation were used for continuous data, and the frequency (events/observations) for dichotomic variables. The mean difference (MD) effect measure and unpaired *t*-test were used to evaluate the improvement in PROMs and functional tests from baseline to the last follow-up. The CI was set at 95% in all the comparisons. Overall values of P < 0.05 were considered statistically significant.

## 3 Results

### 3.1 Study selection

**Fig. 1:**
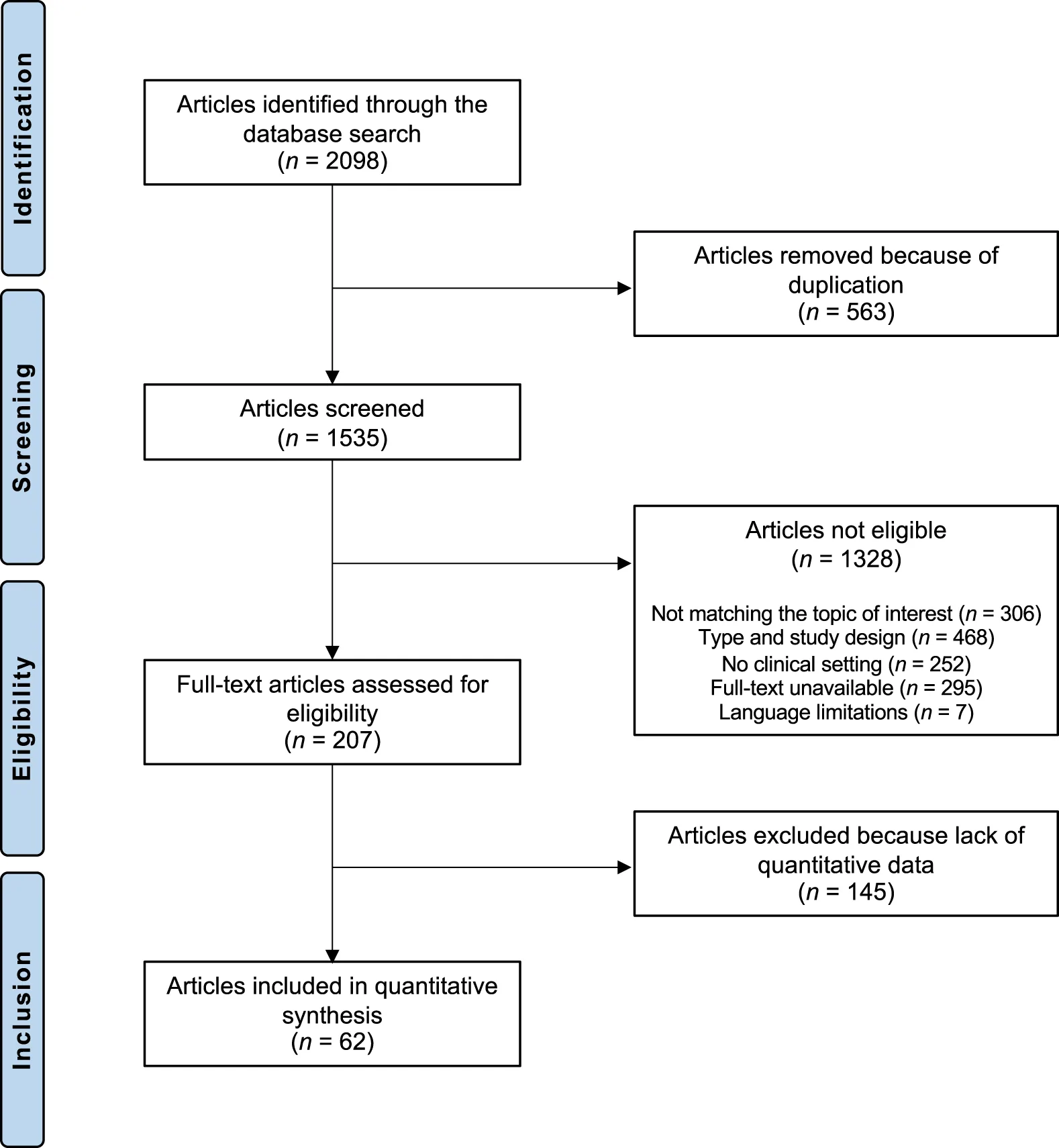
Flow-chart of the literature search.

The systematic literature search yielded a total of 2098 articles. After removal of 563 duplicates, 1535 abstracts were screened for eligibility. Of these, 1328 were excluded, most commonly because the study design did not meet the predefined inclusion criteria (n = 468). Further exclusions applied to studies unrelated to wrist arthrosis (n = 168) or total arthroplasty (n = 138), those conducted outside a clinical setting (n = 252), studies without available full text (n = 295), and a small number published in languages beyond the authors’ proficiency (n = 7). The remaining 207 articles underwent full-text assessment, from which an additional 145 were excluded, given the lack of quantitative outcome data. Ultimately, 62 studies fulfilled all eligibility criteria and were included in the systematic review. The study selection process is illustrated in Fig. 1.

### 3.2 Methodological quality assessment

**Fig. 2:**
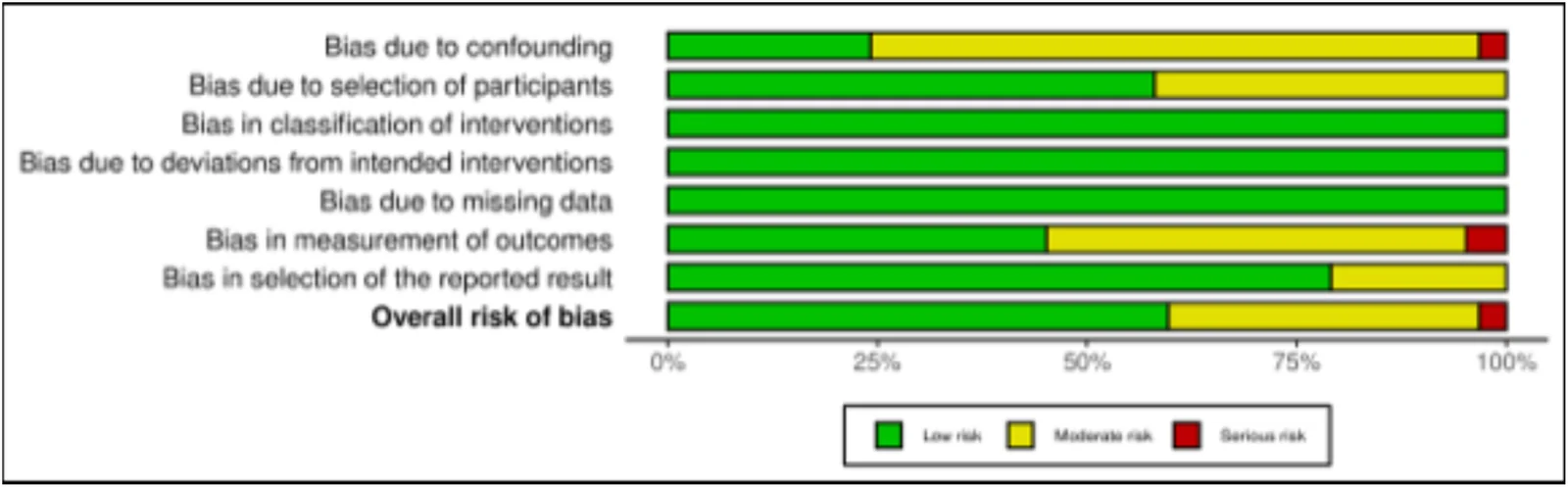
Methodological quality assessment.

The ROBINS-I assessment indicated that most domains were rated as having a low risk of bias (Fig. 2). Bias due to confounding was predominantly judged as low, although several studies were rated as moderate and a small number as serious, reflecting the inherent limitations of non-randomised designs in adequately controlling for potential confounders. The domains of participant selection, classification of interventions, and adherence to intended interventions were consistently judged at low risk, suggesting that recruitment procedures and treatment definitions were generally appropriate and robust. In contrast, outcome-related domains raised more concerns. Missing data and limitations in outcome measurement were frequent, leading to moderate and, in some cases, serious ratings, particularly in studies relying on retrospective designs or subjective assessments. The risk of bias in the selection of reported results was mainly low, but moderate to serious concerns remained in studies lacking pre-registered protocols or prospective outcome definitions. Most studies were assessed as having a low to moderate overall risk of bias, indicating an acceptable methodological quality across the included non-RCTs.

### 3.3 Study characteristics and results of individual studies

Data from 2503 patients comprising 2362 wrists were analysed. Of these, 64.6% (1618 of 2503) were women. The mean age at the time of surgery was 58.4 ± 14.9 years. Table 2 summarises the baseline characteristics and demographic details of the included studies.

### 3.4 Results synthesis

Across all domains, including PROMs, grip strength, and ROM, statistically significant improvements were observed from baseline to the final follow-up (p < 0.001 for each outcome). A comprehensive summary of these results is provided in Table 3.

**Table 3:** Baseline and last follow-up comparison of PROMs and functionality tests results (MD: mean difference; CI: confidence interval; VAS: visual analogue scale; DASH: disability of the arm, shoulder, and hand; PRWE: patient-rated wrist evaluation; RoM: range of motion; FE: flexion and extension).

Endpoint	Baseline	At last follow-up	MD	Standard Error	CI 95%	P
**VAS (mean)**	7.1 ± 1.7	3.0 ± 1.7	−4.1	0.1	[-4.2; −3.9]	<0.01
**DASH (mean)**	55.5 ± 16.7	32.6 ± 13.2	−22.9	0.7	[-24.2; −21.6]	<0.01
**PRWE score (mean)**	60.5 ± 13.6	31.6 ± 18.1	−28.9	1.4	[-31.6; −26.1]	<0.01
**Grip strength kg (mean)**	12.1 ± 8.2	19.1 ± 9.9	7.0	0.5	[6.1; 7.9]	<0.01
**RoM Flexion (°)**	27.6 ± 7.3	32.8 ± 5.7	5.2	0.3	[4.7; 5.7]	<0.01
**RoM Extension (°)**	24.4 ± 7.2	30.8 ± 8.7	6.4	0.3	[5.7; 7.1]	<0.01
**RoM Overall FE (°)**	53.3 ± 15.6	64.6 ± 16.7	11.3	0.6	[10.1; 12.6]	<0.01
**RoM Radial (°)**	6.9 ± 2.9	9.3 ± 4.1	2.4	0.2	[2.0; 2.7]	<0.01
**RoM Ulnar (°)**	15.7 ± 5.2	19.3 ± 6.2	3.8	0.3	[3.2; 4.2]	<0.01
**RoM overall RUD (°)**	22.8 ± 7.4	28.7 ± 7.4	6.0	0.3	[5.3; 6.6]	<0.01
**Pronation (°)**	74.3 ± 7.7	80.3 ± 7.0	6.0	0.4	[5.2; 6.7]	<0.01
**Supination (°)**	68.7 ± 9.9	77.4 ± 8.2	8.7	0.5	[7.8; 9.6]	<0.01
**Overall (°)**	142.8 ± 16.1	156.1 ± 13.4	13.3	0.8	[11.8; 14.7]	<0.01

Among 2503 operated wrists, 924 postoperative complications were reported. Revision surgery was required in 16.3% (408 of 2503) of the cases, while 18.4% (461 of 2503) wrists underwent additional surgical interventions because of the complications (Table 4).

**Table 4:** Rates of postoperative complication, revision and reoperation.

Endpoint	Wrists
**Complications (n)**	924 of 2503 (36.9%)
**Revisions (n)**	408 of 2503 (16.3%)
**Reoperations (n)**	461 of 2503 (18.4%)

## 4 Discussion

According to the main findings of the present meta-analysis, TWA is effective in reducing pain, improving disability scores and ROM of the wrist. The rate of revisions and reoperations was relatively low across all the included studies, but a substantial number of postoperative complications were reported. The findings of this systematic review offer important insights into the clinical utility of TWA and its role in the management of advanced wrist arthrosis. Across more than two thousand wrists included in 62 studies, PROMs and objective functional assessments consistently demonstrated significant improvements from baseline to final follow-up (p < 0.001 for all parameters). The data confirm that TWA represents a motion-preserving alternative to total wrist fusion (TWF), effectively reducing pain and disability while preserving wrist mobility. However, the relatively high overall complication rate (36.9%) and the considerable proportion of cases requiring revision (16.3%) or additional surgery (18.4%) raise ongoing concerns about safety, which continue to restrict its broader use in clinical practice. One of the most striking observations from the included studies is the consistent evidence that TWA provides pain relief and functional recovery. PROMs such as the Disabilities of the Arm, Shoulder and Hand (DASH), Patient-Rated Wrist Evaluation (PRWE), and Visual Analogue Scale (VAS) for pain were significantly improved across all cohorts, regardless of implant type or follow-up duration. ^3^^,^^6^^,^^9^^,^^40^ This matches with earlier comparative research indicating that, although TWF provides dependable pain relief, many patients continue to experience functional limitations that interfere with occupational tasks and recreational activities. ^28^^,^^41^^,^^42^ The ability of TWA to preserve motion, therefore, represents a meaningful advantage, particularly for younger and more active patients who may otherwise experience long-term dissatisfaction following fusion 43, 44, 45. Nonetheless, functional gains must be interpreted alongside the durability of the procedure. In this review, 924 postoperative complications were reported among 2503 wrists, representing a notably high rate even in the context of complex reconstructive procedures. The most common issues involved implant loosening, periprosthetic fracture, tendon-related problems, and infections, in line with previous studies. ^2^^,^^23^ Even though some complications could be managed conservatively, a relevant proportion of them need surgical reintervention, including revision surgery or conversion to fusion. Although TWA offers significant symptomatic improvement, our results indicate that the complication rate continues to represent a major challenge to achieving durable long-term outcomes ^18^^,^^46^, ^47^, ^48^. When outcomes were stratified by implant generation, a clear evolution in performance emerged. Early devices, such as the BIAX prosthesis, were associated with unacceptably high failure rates, with loosening and implant migration as the predominant causes of revision. ^9^^,^^49^^,^^50^ By contrast, fourth-generation implants, including Universal 2, ReMotion, and Maestro, have demonstrated more favourable short-to mid-term results, with improved implant survival and better preservation of motion. ^2^^,^^25^^,^^51^ Their refined implant geometry, fixation technique and material quality have contributed to improving biomechanics and stability. On the other hand, despite these technological improvements, long-term durability data remain unclear, with only a few studies reporting data beyond 10 years or more of follow-up. ^18^^,^^25^^,^^33^^,^^44^^,^^52^ The lack of high-quality prospective registries further limits our understanding of true implant longevity and failure mechanisms. Another important observation concerns the variety of complications among types of implants. For example, the Universal 2 prosthesis has demonstrated promising results for pain relief and function, yet it is associated with frequent loosening and dislocation. ^3^^,^^32^ The ReMotion implant was designed with a modular approach and a cementless attachment intended to prevent stress shielding and allow for bone ingrowth, although the risk of periprosthetic fracture remains. ^53^^,^^54^ The Maestro system was designed to mimic normal wrist motion via a dual-bearing articulation and has shown promising early results but still requires long-term evaluation. ^2^^,^^32^^,^^55^^,^^56^ These design-specific complication profiles highlight the importance of tailoring implant selection to individual patient factors such as bone quality, activity level, and aetiology of arthrosis. ^35^^,^^51^^,^^57^ The present review also emphasises the role of PROMs and functionality tests as primary measures of success in TWA. Traditionally, clinical outcomes focused on radiographic parameters and surgeon-assessed assessments, which often do not reflect the patient's personal and subjective experience of recovery. PROMs such as DASH and PRWE are particularly valuable, giving insights into how wrist arthroplasty impacts activities of daily living and quality of life. ^6^^,^^9^ The included studies suggest that TWA meets patients' expectations regarding pain relief and enhanced wrist ROM, even when complications may occur. ^6^^,^^31^^,^^40^ However, the relationship between PROM improvements and complication rates requires further exploration. It remains unclear why complications such as asymptomatic radiographic loosening lead to patient dissatisfaction. Comparisons between TWA and TWF are crucial to clinical decision-making and patient therapy. ^42^^,^^58^^,^^59^ Several studies have directly compared TWA and TWF outcomes. In general, TWF reported better pain relief, while TWA was better in function ^7^^,^^60^^,^^61^. This conflict is representative of the overall dilemma facing surgeons and patients, deciding between accepting higher complication risks in exchange for functional gains or prioritising long-term predictability, sacrificing the range of motion. A further limitation evident in the current literature is the heterogeneity of study designs and outcome reporting. The majority of included studies were relatively small retrospective case series. This limits statistical power and the ability to generalise findings. Variations in follow-up times and in definitions of complications, revisions, and reoperations introduce additional heterogeneity across studies. Additionally, most included studies lacked a control group, limiting comparisons to TWF or, for that matter, to any intervention. These methodological concerns reinforce the immediate need for reliable prospective multicenter registries and randomised controlled trials to better inform quality evidence on the safety and effectiveness of TWA. Finally, the results of this review have valuable implications for future research as well as clinical practice. Reducing postoperative complications continues to be a priority among researchers, particularly by improving implant fixation, decreasing the risk of periprosthetic fracture, and minimising infection. New advances in 3D printing, patient-specific instrumentation, and biologically-enhanced implants may offer new opportunities to refine the design of prosthetics. Additionally, the establishment of standardised outcome reporting, particularly PROMs and long-term implant survival, will be critical for meaningful comparisons across studies. By addressing these important research gaps, future work will clarify the evidence base and broaden it to inform implant selection, surgical technique, and rehabilitation. All of which ultimately improve the outcomes for patients with advanced wrist arthrosis. In conclusion, this systematic review highlights that TWA can result in considerable improvements in pain relief and functional outcomes relative to preoperative status and suggests that TWA is a meaningful, motion-preserving surgical option for patients with wrist arthrosis compared with wrist fusion. However, the procedure carries a concerning rate of complications, revisions, and reoperations, and our authors believe this remains a concern for patients and surgeons alike. ^7^^,^^48^ Moreover, this variability in outcomes, particularly with respect to implant type, determines the extent of patient selection and surgical planning. Although modern implant designs represent a marked advancement from previous generations, there is a need for additional high-quality studies to support their long-term safety and effectiveness.

## 5 Conclusion

TWA appears to be effective in improving pain and disability scores for patients affected by wrist arthrosis. However, some uncertainty persists regarding the safety profile of the surgical procedure. Future studies should focus on how to reduce postoperative complications. From a clinical perspective, TWA should only be offered in a selective manner to eligible patients after complete and comprehensive preoperative counselling regarding the potential benefits and hazards of the procedure. Shared decision-making plays an important role in the process, while also attending to the individual's personal expectations regarding a TWA procedure, including their occupational requirements and willingness to undergo future surgical interventions. PROMs are a useful mechanism for facilitating patient-centred care and serve as outcome measures. Future research should focus on preventing postoperative complications and improving clinical and functional outcomes. Robust multicenter registries and high-quality comparative studies comparing TWF under clearly defined, predefined conditions with TWA are needed to further clarify implant survival, identify predictors of failure, and provide stronger practice recommendations for surgical management.

## Consent to participate

Not applicable.

## Consent to publish

Not applicable.

## Guardian/patient's consent

Not applicable.

## Ethical approval

This study complies with ethical standards.

## Registration and protocol

The present review was not registered.

## Clinical trial number

Not applicable.

## Availability of data and materials

the datasets generated during and/or analysed during the current study are available throughout the manuscript.

## Credit author statement

GP: conception and design, drafting (revision); FS: data interpretation, drafting (original and revision); LS: literature search, drafting (original and revision); DF, NT: critical revision of the manuscript; JE: supervision, drafting (revision).

## Use of AI

Language editing was performed using Grammarly (Superhuman Platform Inc., San Francisco, CA, USA).

## Funding

The authors received no financial support for the research, authorship, and/or publication of this article.

## Funding statement

The authors received no financial support for the research, authorship, and/or publication of this article.

## Conflict of interest

The authors declare that they have no competing interests in this article.

## Figures and Tables

**Table 2:** Generalities and patient demographics of the included studies.

Author and Year	Journal	Follow-up (years)	Patients (n)	Women (n)	Wrists (n)	Mean age (y)	Prosthesis	Manufacturer
Badge et al., 2016 ^32^	Bone Joint J	4.4	75	59	85	59.0	Universal 2	Integra LifeSciences Corporation, Plainsboro, NJ, USA
Bellemère et al., 2012 ^62^	Chir Main	5.0	25	10	25	60.0	Amandys	Tornier SAS-Bioprofile Grenoble, France
Bidwai et al., 2013 ^27^	Hand Surg	2.8	10	8	11	57.5	Remotion	AVANTA
Biehl et al., 2021 ^63^	Life (Basel)	8.5	32	28	34	56.0	MPW	Link Company™, Hamburg, Germany
Boeckstyns et al., 2013 ^28^	Acta Orthop	6.5	60	48	65	58.0	Remotion	AVANTA
Bosco et al., 1994 ^64^	J Arthroplasty	8.6	16	12	18	54.0	Volz	Howmedica Company, Rutherford, NJ
Cai et al., 2023 ^65^	Front Bioeng Biotechnol	1.6	14	7	14	45.2	3D-printed microporous titanium artificial wrist prosthesis (3DMT-Wrist system)	
Chevrollier et al., 2016 ^66^	Acta Orthop Belg	5.2	16	9	17	59.0	Universal; Remotion	KMI, San Diego, CA, USA; BI, Morrisville, PA,USA
Cobb et al., 1996 ^67^	J Hand Surg Am	6.5	52	45	64	57.5	Biaxial	DePuy, Warsaw, IN, USA
Comstock et al., 1988 ^68^	J Hand Surg Am	6.0	19		20		Swanson	Wright Medical, Memphis, TN, USA
Cooney et al., 2012 ^29^	J Wrist Surg	6.0	39	28	46	57.0	Biaxial, Universal 2, Re-motion	DePuy, Warsaw, IN, USA; Integra LifeSciences Corporation, Plainsboro, NJ, USA; AVANTA
Courtman et al., 1990 ^69^	J Hand Surg Br	2.8	26	19	26	56.8	Biaxial	Small Bone Innovations Inc; Morrisville, PA, USA
Divelbiss et al., 2002 ^70^	J Hand Surg Am	1.0	19	19	22	48.1	Universal 1	KMI, San Diego, CA
Ekroth et al., 2012 ^71^	J Wrist Surg	17.8	9		12	68.3	Biaxial; Volz	Biomet, Warsaw, IN; Stryker, Mahwah, NJ
Ferreres et al., 2011 ^72^	J Hand Surg Am	5.5	21	14	21	54.0	Universal; Universal 2	KMI San Diego, CA; Integra LifeSciences Corporation, Plainsboro, NJ, USA
Figgie et al., 1990 ^73^	J Hand Surg Am	9.0	26	23	35	58.0	Trispherical TWP	
Fischer et al., 2020 ^52^	J Hand Surg Am	10.0	124	106	136	60.0	Biax, Remotion, UWP-2, Maestro	DePuy Orthopaedics, Warsaw, IN; Stryker, Kalamazoo, MI; Integra Life Sciences, Plainsboro, NJ; Biomet, Warsaw, IN
Friedel et al., 2008 ^74^	Obere Extremität	3.0	9	6	9	53.0	Universal 1; Universal 2	KMI San Diego, CA; Integra LifeSciences Corporation, Plainsboro, NJ, USA
Froschauer et al., 2019 ^75^	J Hand Surg Eur Vol	7.0	38	16	39	56.0	Re-Motion TWA	Stryker, Kalamazoo, MI, USA
Gendera et al., 2020 ^76^	Acta Orthop Belg	7.5	14	8	14	61.0	Universal 2 prosthesis, Integra	Integra LifeSciences Corporation, Plainsboro, NJ, USA
Giacalone et al., 2017 ^77^	Plast Reconstr Surg	2.8	25	2	25	58.0	Resurfacing Capitate Pyrocarbon Implant	Tornier, Grenoble, France
Gil et al., 2017 ^25^	Clin Orthop Relat Res	9.0	39	36	39	56.0	Universal2	Integra LifeSciences Corporation, Plainsboro, NJ, USA
Giwa et al., 2017 ^36^	J Hand Surg Asian Pac Vol	5.5	23	8	25	61.0	Motec cementless modular metal-on-metal ball-and-socket wrist arthroplasty	Swemac Orthopaedics AB, Linköping, Sweden
Goubier et al., 2011 ^78^	Tech Hand Up Extrem Surg	2.5	7	1	7	49.0	Pyrocarbon prothesis RCPI	Resurfacing Capitate Pyrocarbon Implant Prosthesis, Tornier SAS, Etablissements Bioprofile, France
Herzberg et al., 2012 ^53^	J Wrist Surg	2.0	210	142	215	63.0	Ball and socket ReMotion TWA	Small Bone Innovation, Morristown, PA
Holzbauer et al., 2022 ^79^	Life (Basel)	6.2	32	12	32	59.2	ReMotion Total Wrist System	Stryker, Kalamamazoo, zoo, Michigan
Honecker et al., 2019 ^44^	J Wrist Surg	6.0	22	18	23	55.0	Re-motion	Small Bone Innovation, Morristown, PA
Jolly et al., 1992 ^80^	J Hand Surg Am	6.0	23	18	23	47.0	Swanson	Wright Medical, Memphis, TN, USA
Kennedy et al., 2018 ^81^	J Hand Surg Eur Vol	7.1	46	33	48	63.5	Universal 2	Integra LifeSciences Corporation, Plainsboro, NJ, USA
Kistler et al., 2005 ^82^	J Hand Surg Am	10.0	25	20	27	50.0	Swanson, silicone implant	Wright Medical, Memphis, TN, USA
Kretschmer et al., 2007 ^83^	Handchir Mikrochir Plast Chir	2.6	42	13	42	53.0	BIAX	DePuy Orthopedics, Inc. Warsaw, IN
Krukhaug et al., 2011 ^84^	Acta Orthop	16.5	189	133	189	64.0	Biaxial; Elos 1, 2, 3; Gibbon	DePuy, Warsaw, IN; Swemac Orthopaedics AB, Linkoping, Sweden
Lestienne et al., 2021 ^85^	Hand Surg Rehabil	5.3	23	18	28	55.7	Amandys pyrocarbon	Wright-Medical, Tornier, Montbonnot Saint Martin, France
Levadoux et al., 2003 ^86^	J Hand Surg Am	3.9	25	5	28	62.5	Destot	
Marcuzzi et al., 2014 ^87^	J Hand Surg Eur Vol	2.8	35	9	35	54.0	RCPI (Resurfacing capitate pyrocarbon implant)	Tornier SAS, Etablissements Bioprofile, France
Marie et al., 2021 ^88^	Hand Surg Rehabil	3.4	13	6	13	43.8	Amandys	Wrigh, Grenoble, France
Matsui et al., 2019 ^89^	J Hand Surg Am	5.6	20	15	20	64.0	Matsui Semiconstrained TWA	
Menon et al., 1998 ^90^	J Arthroplasty	6.7	31	24	37	58.1	Universal 1	Kinetikos Medical Inc.,4115 Sorrento ValleyBlvd., San Diego, CA, USA
Meuli et al., 1995 ^91^	J Hand Surg Am	4.5	45	35	50	55.0	MWP III (Melui Wrist Prosthesis, third reised implant)	Sulzer Orthopaedics Ltd., Winterthur, Switzerland
Morapudi et al., 2012 ^92^	J Orthop Surg (Hong Kong)	3.1	19	14	19	62.0	Universal 2	Integra LifeSciences Corporation, Plainsboro, NJ, USA
Nydick et al., 2012 ^31^	J Hand Surg Am	2.3	22		23	63.0	Maestro	Biomet, Warsaw, IN, USA
Pfanner et al., 2017 ^93^	J Wrist Surg	6.9	22	20	23	54.9	Universal 2	"Integra Life Sciences, Plainsboro, NJ, USA; previously manufactured by Kinetikos Medical Inc."
Pierrart et al., 2012 ^94^	Chir Main	1	11	4	11	55.2	Amandys	Tornier SAS-Bioprofile Grenoble, France
Radmer et al., 2003 ^95^	J Hand Surg Am	4.3	40	32	40	48.0	APH prosthesis	Implant-Service Vertriebs-GmbH, Hamburg, Germany
Rahimtoola et al., 2003 ^96^	Scand J Plast Reconstr Surg Hand Surg	4.0	27	18	29	59.0	RWS Prosthesis	HowmedicaTM, Pfizer Hospital Products Group, The Netherlands
Rahimtoola et al., 2004 ^97^	J Hand Surg Br	1.6	30	26	32	60.0	Micromed, Germany	
Reigstad et al., 2017 ^33^	J Hand Surg Am	8.0	57	17	57	52.0	Motec	Swemac Orthopaedics AB, Linköping, Sweden
Reigstad et al., 2012 ^98^	J Bone Joint Surg Br	3.2	30	10	30	52.0	Motec cementless modular metal-on-metal ball-and-socket wrist arthroplasty	Swemac Orthopaedics AB, Linköping, Sweden
Rizzo et al., 2003 ^99^	J Hand Surg Am	6.0	14	10	17	58.6	Biaxial	DePuy Orthopedics, Inc., Warsaw, IN
Rossello et al., 2022 ^100^	Indian J Orthop	4.0	12	6	12	61.7	Freedom	Intefra Lifesciences, Princeton, USA
Sagerfors et al., 2015 ^16^	J Hand Surg Am	7.0	219	186	219	60.0	Biax; Remotion; Universal 2; Maestro	DePuy Orthopedics, Warsaw, IN; Stryker, Kalamazoo, MI; Integra LifeSciences Corporation, Plainsboro, NJ, USA; Biomet, Warsaw, IN
Schill et al., 2001 ^101^	Handchir Mikrochir Plast Chir	9.7	72	71	82	56.9	Swanson	Wright Medical, Memphis, TN, USA
Stegemann et al., 2005 ^102^	Rheumatol Int	2.1	14	10	16	53.0	Biaxial	DePuy Orthopedics, Inc., Warsaw, IN
Strunk et al., 2009 ^103^	Handchir Mikrochir Plast Chir	5.3	15		15	54.0	Meuli; BIAX; Universal 2	Integra LifeSciences Corporation, Plainsboro, NJ, USA
Takwale et al., 2002 ^104^	J Bone Joint Surg Br	4.3	63	62	66	59.3	Biaxial	DePuy Orthopaedics Inc, Leeds, UK
Tanwin et al., 2021 ^57^	J Wrist Surg	7.0	59	35	63	58.0	Amandys	Wright Medical-Tornier, SAS, Bioprofile, Grenoble, France
Truss et al., 2023 ^105^	J Wrist Surg	15.6	19	14	21	63.0	Universal 2	Integra LifeSciences Corporation, Plainsboro, NJ, USA
van Harlingen et al., 2011 ^106^	Acta Orthop	6.0	36	28	40	54.0	Biaxial, designed by Beckenbaugh	DePuy Orthopaedics Inc, Leeds, UK
van Winterswijk et al., 2010 ^107^	Open Orthop J	3.8	15	11	17	64.0	Universal 1	KMI, San Diego, CA
Ward et al., 2011 ^48^	J Bone Joint Surg Am	7.3	15	15	19		Universal 1	KMI, San Diego, CA
Zhang et al., 2023 ^108^	Front Bioeng Biotechnol	1.6	7		7		3D printed tantalum prosthesis.	
Zijlker et al., 2019 ^19^	J Wrist Surg	11.0	25	16	26	49.0	Universal2	Integra LifeSciences Corporation, Plainsboro, NJ, USA
